# Maternal SARS-CoV-2 infection during pregnancy and child neurodevelopmental outcomes: A systematic review and meta-analysis of observational studies

**DOI:** 10.1016/j.pmedr.2026.103508

**Published:** 2026-05-23

**Authors:** Ryan Whalley, Amalie Primdal, Iain Hardie, Zhuoni Xiao, Bonnie Auyeung

**Affiliations:** a Department of Psychology, School of Philosophy, Psychology and Language Sciences, University of Edinburgh, Edinburgh, United Kingdom; b Division of Psychiatry, Centre for Clinical Brain Sciences, University of Edinburgh, Edinburgh, United Kingdom

## Abstract

**Objective:**

To assess: (a) associations between prenatal SARS-CoV-2 exposure and early childhood neurodevelopmental outcomes, and (b) whether these varied by infection trimester.

**Methods:**

We conducted a systematic review and meta-analysis of observational studies modelling the neurodevelopmental outcomes of children prenatally exposed to SARS-CoV-2 compared to unexposed children. PubMed and PsychInfo were systematically searched from inception to March 2025. Primary analysis was random-effects modelling of overall associations. Secondary analysis assessed variation by trimester via standardised mean differences (SMD).

**Results:**

11 observational studies were included. Overall, there was no statistically significant association between prenatal SARS-CoV-2 exposure and early childhood neurodevelopmental outcomes, both before (odds ratio: 1.08; 95% confidence intervals: 0.82, 1.42) and after (odds ratio: 0.98; 95% CI: 0.81, 1.17) excluding high variance studies. First trimester prenatal SARS-CoV-2 exposure was associated with poorer neurodevelopmental scores (3.87; 95% CI: 3.19, 4.55) than prenatal exposure in the second trimester (6.07; 95% CI: 5.11, 7.03) or third trimester (6.31; 95% CI: 5.54, 7.09). However, this analysis was limited to three studies only, with high heterogeneity between studies.

**Conclusions:**

Prenatal SARS-CoV-2 exposure is not associated with early childhood neurodevelopmental outcomes. Future research should keep monitoring associations as more data becomes available. More robust research is required on trimester-specific associations.

## Highlights

• Prenatal SARS-CoV-2 exposure is not significantly associated with early child development.
• Future research should continue to monitor this as children age and more data becomes available.
• Moreover, robust research is still required on variations in associations by trimester.

## 1 Introduction

Coronavirus disease (COVID-19), caused by severe acute respiratory syndrome coronavirus 2 (SARS-CoV-2) infection, is a highly transmissible viral illness with significant global health implications. During the COVID-19 pandemic, those who were pregnant were identified as being at higher risk of severe COVID-19 disease (Allotey et al., 2020). As well as impacting maternal ill-health, there were also concerns during the pandemic that maternal SARS-CoV-2 infections during pregnancy may have potential implications for unborn children, including that prenatal SARS-CoV-2 exposure may have long-term effects on infant neurodevelopment.

In particular, the prenatal period is known to be a critical period for neurodevelopment (Estes and McAllister, 2016), and there is growing recognition that a mother's immune response to infection, known as maternal immune activation, may potentially impact child neurodevelopment (Han et al., 2021). As such, there have been concerns that prenatal SARS-CoV-2 exposure may have implications for child neurodevelopment via the mechanism of maternal immune activation (Shook et al., 2022). Severe forms of other non-SARS-CoV-2 infections (including bacterial, viral and other infections) during pregnancy have previously been found to be associated with child neurodevelopmental outcomes (Hardie et al., 2025a; Green et al., 2018; Lee et al., 2020; Kwok et al., 2022), suggesting it is possible that the same could be true for SARS-CoV-2 infections during pregnancy. In addition to this, SARS-CoV-2 infection during pregnancy itself has been found to be associated with increased risk of adverse neonatal outcomes like preterm birth (Lindsay et al., 2023), which can in turn be associated with later neurodevelopmental outcomes (Hee Chung et al., 2020).

Due to the recency of the COVID-19 pandemic, evidence on maternal exposure to SARS-CoV-2 during pregnancy and child neurodevelopment remains limited, and potential impacts are not fully understood. Nonetheless, a growing number of observational studies on this topic are being conducted internationally. To date, there are now five systematic or rapid reviews conducting meta-analysis on associations between prenatal SARS-CoV-2 exposure and neurodevelopmental and other outcomes (Pinheiro et al., 2023; Hessami et al., 2022; Jackson et al., 2024a; Sturrock et al., 2023; Rizzo et al., 2025). One recent review, Jackson et al. (2024a) found no consistent association between antenatal or neonatal exposure to SARS-CoV-2 and child neurodevelopment up to age 12 months (Jackson et al., 2024a). Similarly, Hessami et al. (2022) found that overall neurodevelopment in the first year of life was not changed by prenatal SARS-CoV-2 exposure (Hessami et al., 2022), whilst Rizzo et al. (2025) had mixed findings but concluded that their analysis did not suggest a causal relationship between prenatal exposure to SARS-CoV-2 infection or COVID-19 vaccination and adverse neurodevelopmental outcomes. In contrast, the other two reviews did find evidence of associations between maternal exposure to SARS-CoV-2 infection and some adverse neurodevelopmental outcomes (Pinheiro et al., 2023; Sturrock et al., 2023). All five reviews emphasise, however, the scarcity of studies, small sample sizes, and the limited follow-up beyond infancy, making it difficult to draw conclusions on causality. Collectively, they highlight the need for larger, longitudinal cohorts with extended neurodevelopmental follow-up (Pinheiro et al., 2023; Hessami et al., 2022; Jackson et al., 2024a; Sturrock et al., 2023).

These existing systematic reviews have a fairly wide-ranging focus, covering not just prenatal SARS-CoV-2 exposure but also COVID-19 vaccination exposure, and involving a range of outcomes beyond neurodevelopmental outcomes. Moreover, a number of additional observational studies on associations between prenatal SARS-CoV-2 exposure and child neurodevelopment, including large cohort studies, have been conducted since previous meta-analyses were published. As such, we conducted a new systematic review and meta-analysis of studies specifically investigating associations between prenatal exposure to SARS-CoV-2 and childhood neurodevelopment. Given that previous research has found that associations between non-SARS-CoV-2 infections during pregnancy (including other forms of viral, bacterial or other infections) and child neurodevelopmental outcomes can vary by trimester (Hardie et al., 2025a; Kwok et al., 2022), we also focus on whether there may be variation in associations by trimester of SARS-CoV-2 infection.

Specifically, we drew on robust observational studies using quantifiable and standardised neurodevelopmental measures to address the following research questions:

**RQ1**: Do existing observational studies suggest that maternal SARS-CoV-2 infections during pregnancy are associated with early childhood neurodevelopmental outcomes?

**RQ2**: Do existing observational studies suggest that associations between maternal SARS-CoV-2 infections during pregnancy and early childhood neurodevelopmental outcomes vary by the trimester of infection exposure?

## 2 Methods

### 2.1 Registration

This systematic review and meta-analysis was registered through the International Prospective Register of Systematic Reviews (PROSPERO, registration number: CRD42025641913), following PRISMA 2020 guidelines (Page et al., 2021). A PRISMA 2020 checklist is provided in Supplementary Appendix A.

### 2.2 Data sources and search strategy

PubMed and PsychInfo databases were systematically searched from inception to March 2025. Searches were for peer-reviewed observational studies examining associations between maternal SARS-CoV-2 infections during pregnancy and early child neurodevelopmental outcomes. The key terms used for each database are provided in Supplementary Appendix B.

### 2.3 Eligibility criteria, measures and study selection

Studies meeting the following inclusion criteria were included: (1) were observational studies (made up of cohort, cross-sectional or case control studies) examining associations between maternal SARS-CoV-2 infection during pregnancy and child neurodevelopmental outcomes, (2) compared exposed children to a comparison group of unexposed children, and (3) used a child neurodevelopmental outcome measure that was quantifiable and standardised.

The following types of studies were excluded: (1) those not written in English, (2) those examining other non-SARS-CoV-2 infections alongside SARS-CoV-2 infections, and (3) those examining children below one month old only (as these children had not yet reached key neurodevelopmental milestones).

The neurodevelopmental outcome measures used in studies included validated developmental screening tools like Ages and Stages Questionnaire 3rd edition (ASQ-3), neurodevelopmental disorders identified via International Classification of Diseases 10th revision (ICD-10), and other quantifiable and standardised measures like developmental concerns identified during routine child health reviews.

With regard to exposure measures, we included studies which measured confirmed SARS-CoV-2 infections (via laboratory diagnosis, clinical records or viral testing) during pregnancy, and compared prenatally exposed children to a comparison group of prenatally unexposed children. In studies which also examined trimester-specific prenatal SARS-CoV-2 infections, we used these as additional exposure measures.

The search strategy outlined above was used to identify papers from PubMed and PsychInfo. Papers were then screened to remove duplicates, and remaining papers were filtered based on title and abstract relevance. Relevant studies were then selected for further full text review, and those which met all of our eligibility criteria were included in the systematic review and meta-analysis. Additional studies not included in our PubMed and PsychInfo searches, but which met all eligibility criteria, were identified through citation searching of identified papers and through searching for relevant studies via external websites and organisations. The article screening process was carried out independently by two researchers (RW and AP) with any disagreements being discussed and resolved with a third researcher (BA).

### 2.4 Data extraction and quality assessment

Two researchers (RW and AP) independently conducted data extraction using a standardised data extraction sheet. For each study included in the meta-analysis, data was extracted on study location, study period, study design, sample size (exposed and unexposed), exposure timing by trimester (where applicable), age of neurodevelopmental assessment, method of neurodevelopmental assessment, and study result. In studies including neurodevelopmental assessment of children at multiple ages, we extracted most recent study results (oldest age of assessment) only, to prevent confirmatory bias. Following on from data extraction, the methodological quality of each study was assessed using the standard Newcastle-Ottowa Scale (NOS) for cohort studies (Wells et al., 2021). This assessment scale measures the quality of non-randomised studies across three domains: (1) selection, (2) comparability, and (3) outcome. Up to four points (or ‘stars’) are awarded for selection, up to two for comparability and up to three for outcome. This gives a total score with a maximum of nine points, with 0–3 points indicating very high risk of study bias, 4–6 points indicating high risk of bias and 7–9 points indicating low risk of bias. Full details of the NOS scoring system are provided in Supplementary Appendix C.

### 2.5 Data synthesis and Meta-analysis

Primary analysis examined associations between maternal SARS-CoV-2 infection during pregnancy and child neurodevelopmental outcomes. Here, odds ratios and 95% confidence intervals (CI) from each of the studies were analysed using a random-effects model with restricted maximum likelihood estimation to account for variability between studies (Viechtbauer, 2010). Odds ratios were taken from measures of associations between maternal SARS-CoV-2 infection during pregnancy and overall child neurodevelopment provided in the studies. In cases where studies reported results for multiple outcomes (for example, looked at multiple neurodevelopmental domains) without providing an overall measure, results were pooled to generate an overall odds ratio and ensure consistency. Meta-analysis was firstly carried out with all studies included. Additional meta-analysis was then conducted excluding any studies with high levels of variance if there was high heterogeneity (to avoid these studies potentially inflating the results). Heterogeneity was assessed using a two-sided I^2^ test, with I^2^ < 0.50 indicating significant levels of heterogeneity. Cochrane's Q test was used to assess reduction in heterogeneity when high variance studies were excluded. Publication bias was also evaluated via a funnel plot.

Secondary analysis examined whether associations between maternal SARS-CoV-2 infections during pregnancy and child neurodevelopmental outcomes varied by trimester of infection exposure. Here, we only included studies with trimester-specific data. This analysis involved exposed children only. There was no unexposed comparison group. As such, standardised mean differences (SMDs) based on developmental scores among those exposed in different trimesters were calculated. These were calculated from the reported means and standard deviations per trimester. While these SMDs do not reflect differences between exposed and unexposed infants, they enabled developmental scores from different assessment tools to be standardised and placed on a scale enabling comparison between those exposed in different trimesters. This facilitated a descriptive analysis of whether associations between prenatal SARS-CoV-2 exposure and neurodevelopmental outcomes differed by trimester of exposure.

As this study was a systematic review of previously published analyses, ethical approval was not required. All analysis was conducted in the R environment version 4.3.1, using the ‘metafor’ and ‘meta’ packages.

## 3 Results

### 3.1 Study selection

**Fig. 1:**
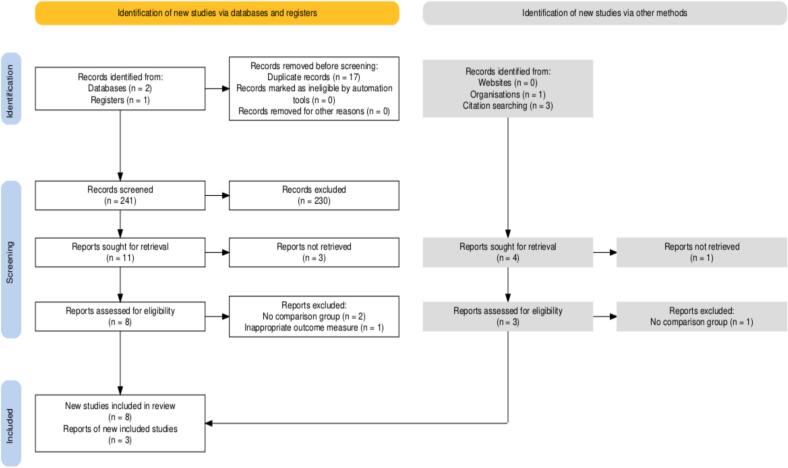
PRISMA flow diagram of study selection for studies on associations between prenatal SARS-CoV-2 exposure and early childhood neurodevelopmental outcomes identified through searches from database inception to March 2025.

A total of 258 papers were identified from the PubMed and PsychInfo databases, of which 241 remained after removing duplicates. We additionally identified four studies via citation searching of identified papers and one from an organisation (The University of Edinburgh). After filtering studies based on title and abstract relevance, 15 studies were selected for further full text review. 11 of these met all eligibility criteria and were included in the meta-analysis. This full process is outlined in Fig. 1.

### 3.2 Study characteristics

Full details of data extracted on study characteristics, study results and NOS quality assessment scores are provided in Table 1. To summarise, a total of 12 cohorts from 11 studies were included in our review, with two distinct cohorts being analysed separately from each other from the Firestein et al. (2024) study. All studies were published between 2022 and 2025. Of the 11 studies, five were from the United States, two were from the United Kingdom, one was from Brazil, one was from Spain, one was from both the United States and Brazil and one was from both the United States and the territory of Puerto Rico.

**Table 1:** Characteristics and quality assessment scores for included studies on associations between prenatal SARS-CoV-2 exposure and early childhood neurodevelopmental outcomes, included studies published 2022–2025.

Study authors and year	Study country	Study period	Study design	Sample size (exposed and unexposed)	Trimester of exposure	Age of assessment	Method of outcome assessment	Study result (odds ratio and 95% CI)	NOS quality assessment score
(Ayesa-Arriola et al., 2023)	Spain	Dec 2020-Nov 2021	Cohort study	Exposed: 21 Unexposed: 21	First: 3 Second: 8 Third: 10	6 weeks	NBAS	1.16 (0.52, 2.61)	7
(Edlow et al., 2022)	United States	Mar 2020- Sep 2020	Retrospective cohort study	Exposed: 190 Unexposed: 6896	N/A	12 months	ICD-10	1.68 (0.81, 3.45)	8
(Edlow et al., 2023)	United States	Mar 2020- May 2021	Retrospective cohort study	Exposed: 218 Unexposed: 4609	N/A	12 months	ICD-10	1.27 (0.88, 1.83)	9
(Fajardo-Martinez et al., 2024)	United States/Brazil	Apr 2020- Dec 2022	Cohort study	Exposed: 128 Unexposed: 128	N/A	11–30 months	Bayley-III	6.52 (1.43, 29.74)	7
(Firestein et al., 2023b)	United States	Mar 2021- Jun 2022	Cohort study	Exposed: 112 Unexposed: 258	First: 14 Second: 41 Third: 36	5–11 months	DAYC-2	0.95 (0.79, 1.13)	7
(Firestein et al., 2024)	United States	Jan 2018- Sep 2021	Cohort study	Exposed: 130 Unexposed: 997	N/A	16–18 months	M-CHAT-R	0.40 (0.22, 0.68)	8
(Firestein et al., 2024)	United States	Feb 2020- Sep 2021	Prospective longitudinal cohort study	Exposed: 101 Unexposed: 201	N/A	18 months	M-CHAT-R	0.51 (0.24, 1.04)	8
(Hardie et al., 2025b)	United Kingdom	May 2020- Sep 2021	Retrospective cohort study	Exposed: 1631 Unexposed: 23,288	N/A	13–15 months	Developmental concerns	1.16 (0.78, 1.71)	8
(Jackson et al., 2024b)	United Kingdom	Oct 2021- Jan 2023	Prospective cohort study	Exposed: 96 Unexposed: 243	N/A	21–32 months	ASQ-3	1.19 (0.55, 2.56)	7
(Jaswa et al., 2024)	United States/Puerto Rico	May 2020- August 2021	Cohort study	Exposed: 151 Unexposed: 1371	N/A	24 months	ASQ-3	0.99 (0.67, 1.46)	8
(Santos et al., 2024)	Brazil	Apr 2020- Jul 2021	Prospective cohort study	Exposed: 69 Unexposed: 68	N/A	12 months	IMCI	3.64 (1.37, 9.67)	7
(Shuffrey et al., 2022)	United States	Mar 2020- Dec 2020	Cohort study	Exposed: 114 Unexposed: 141	First: 25 Second: 54 Third: 35	6 months	ASQ-3	1.00 (0.82, 1.23)	6

*Notes: Firestein et al. (2024) included two distinct cohorts within the same study. Odds ratios are based on overall measures provided in the studies. In cases where studies reported results for multiple outcomes without providing an overall measure we pooled results to generate an overall odds ratio, to ensure consistency. Sample sizes for exposed and unexposed groups reflect those included in the meta-analysis for the relevant outcome, and therefore may not correspond to the full sample sizes of each study. Abbreviations of outcome assessments: ASQ-3: Ages and Stages Questionnaire 3rd edition, BAYLEY III: Bayley Scales of Infant and Toddler Development 3rd edition, DAYC-2: Developmental Assessment of Young Children 2nd edition, NBAS: Neonatal Behavioural Assessment Scale, M-CHAT-R: Modified Checklist for Autism in Toddlers revised, ICD-10: International Classification of Diseases 10th revision, IMCI: Integrated Management of Childhood Illness.

The 11 studies included a total of 3767 children prenatally exposed to SARS-CoV-2 infection (where child sex data was available, this included 1913 male children and 1826 female children) and 52,151 unexposed children (where child sex data was available, this included 26,570 male children and 25,337 female children). All children were aged between 1 and 38 months. Three of the 11 studies, by Shuffrey et al. (2022), Firestein et al. (2023a) and Ayesa-Arriola et al. (2023), included comparable data on trimester of infection, covering 42 children prenatally exposed in the first trimester of pregnancy, 103 children prenatally exposed in the second trimester of pregnancy and 81 children prenatally exposed in the third trimester of pregnancy.

To assess neurodevelopment, studies used a variety of measures. Three studies used the Ages and Stages Questionnaire 3rd edition (ASQ-3), one used the Developmental Assessment of Young Children 2nd edition (DAYC-2), one used the Modified Checklist for Autism in Toddlers revised (M-CHAT-R), two used the International Classification of Diseases 10th revision (ICD-10), one used the Neonatal Behavioural Assessment Scale (NBAS), one used the Bayley Scales of Infant and Toddler Development 3rd edition (Bayley-III), one used the Integrated Management of Childhood Illness (IMCI) and one used overall child developmental concerns identified during routine child health reviews (here, children were formally assessed by trained health visitors for any developmental concerns regarding speech–language–communication, problem solving, gross motor, personal–social, and emotional–behavioural development). With the exception of Shuffrey et al. (2022) (Shuffrey et al., 2022), all studies had NOS quality assessment scores that indicated a low risk of bias. Additional information on NOS quality assessment scores for selection, comparability and outcome are provided in Supplementary appendix D.

### 3.3 Primary meta-analysis

Results from the primary meta-analysis including all 12 cohorts from all 11 studies are provided in Table 2. These results are shown in table form rather than via forest plot due to high variance in some models leading to very wide CIs (making forest plots difficult to interpret). The forest plot is however provided in Supplementary Appendix E. Overall, the results of this modelling suggested that there were no statistically significant associations between maternal SARS-CoV-2 infections during pregnancy and odds of negative early childhood neurodevelopmental outcomes (odds ratio: 1.08; 95% CI: 0.82, 1.42). However, high heterogeneity was observed (I^2^ = 77%).

**Table 2:** Meta-analysis random-effects modelling results on associations between maternal SARS-CoV-2 infections during pregnancy and early childhood neurodevelopmental outcomes (including high-variance studies), included studies published 2022–2025.

Study	Odds Ratio (95% CI)
Ayesa et al. (2023)	1.16 (0.52, 2.61)
Edlow et al. (2022)	1.68 (0.81, 3.47)
Edlow et al. (2023)	1.27 (0.88, 1.83)
Fajardo-Martinez et al. (2024)	6.52 (1.43, 29.73)
Firestein et al. (2023)	0.95 (0.79, 1.13)
Firestein et al. (2024a)	0.40 (0.23, 0.70)
Firestein et al. (2024b)	0.51 (0.25, 1.06)
Hardie et al. (2025)	1.15 (0.80, 1.65)
Jackson et al. (2024)	1.19 (0.55, 2.58)
Jaswa et al. (2024)	0.99 (0.67, 1.46)
Santos et al. (2024)	3.64 (1.37, 9.67)
Shuffrey et al. (2022)	1.00 (0.82, 1.23)
Random-effects model	1.08 (0.82, 1.42)
Heterogeneity	I^2^ = 77%

**Fig. 2:**
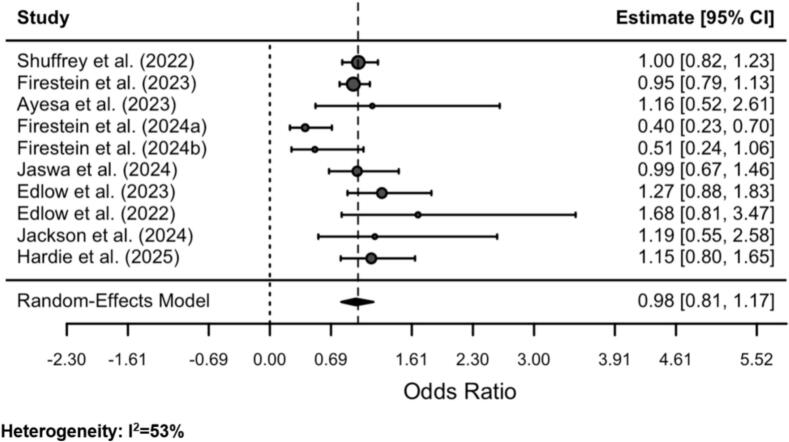
Meta-analysis random-effects modelling results on associations between maternal SARS-CoV-2 infections during pregnancy and early childhood neurodevelopmental outcomes (excluding high-variance studies), included studies published 2022–2025.

Due to this high heterogeneity, we repeated the modelling but with studies with high variance, specifically Fajardo-Martinez et al. (2024) (Fajardo-Martinez et al., 2024) and Santos et al. (2024) (Santos et al., 2024), excluded (to avoid these studies potentially inflating the results). The results of this additional modelling are provided in Fig. 2. To ease interpretation, these results are provided via forest plot. Results were consistent with the modelling including the high variance studies. There were no statistically significant associations between maternal SARS-CoV-2 infections during pregnancy and odds of negative early childhood neurodevelopmental outcomes (odds ratio: 0.98; 95% CI: 0.81, 1.17). Heterogeneity was substantially reduced in this model, although it was still at a moderate level (I^2^ = 53%). Funnel plots for the analysis with and without the inclusion of high variance are provided in Supplementary Appendix F and Supplementary Appendix G. These showed low risk of publication bias when high variance studies were excluded.

### 3.4 Secondary meta-analysis

**Fig. 3:**
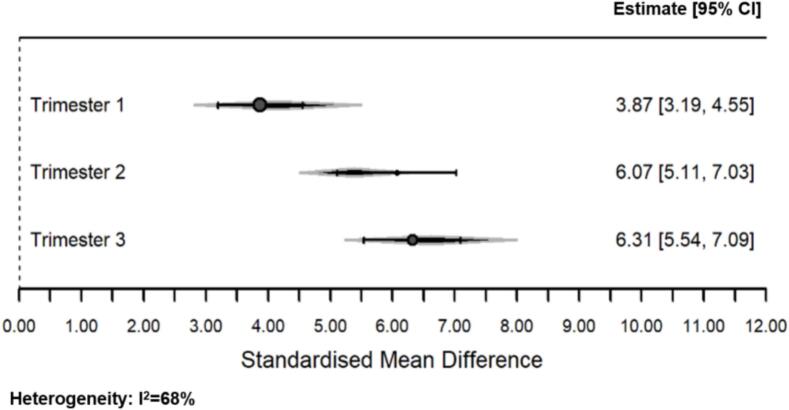
Meta-analysis results on variations in associations between maternal SARS-CoV-2 infections during pregnancy and early childhood neurodevelopmental outcomes by trimester of exposure, included studies published 2022–2023.

Results from the secondary meta-analysis are provided in Fig. 3. Three studies (those which had comparable data on trimester of exposure, Shuffrey et al. (2022) (Shuffrey et al., 2022), Firestein et al. (2023) (Firestein et al., 2023a), and Ayesa-Arriola et al. (2023) (Ayesa-Arriola et al., 2023)) were included in this analysis, incorporating three different assessment methods (ASQ-3, NBAS and DAYC-2). Higher SMDs indicate better neurodevelopmental outcome scores, while lower SMDs indicate poorer developmental outcome scores. The results suggested that first trimester SARS-CoV-2 exposure was associated with poorer total neurodevelopmental scores (3.87; 95% CI: 3.19, 4.55) than second (6.07; 95% CI: 5.11, 7.03) or third (6.31; 95% CI: 5.54, 7.09) trimester exposure. Heterogeneity was high (I^2^ = 68%).

## 4 Discussion

This systematic review and meta-analysis examined whether maternal SARS-CoV-2 infections during pregnancy are associated with early childhood neurodevelopmental outcomes, and whether this varied by trimester of infection exposure. Based on 11 observational studies, our primary analysis suggests no significant association exists between prenatal exposure to SARS-CoV-2 infection and neurodevelopmental outcomes. This is reassuring evidence, and indicates that SARS-CoV-2 infection during pregnancy, and its associated maternal immune activation, does not appear to be linked to early child neurodevelopment. However, given the recency of the COVID-19 pandemic, our analysis only included children aged between 1 and 38 months. Therefore, our findings should be treated with caution since developmental concerns often emerge when children are older than this. It is also important to note that our meta-analysis examined maternal SARS-CoV-2 infections only, and it is possible that broader pandemic-related factors such as environmental stressors (for example, lockdown measures, reduced social interaction and parental anxiety) may be associated with child developmental outcomes more than prenatal exposure to SARS-CoV-2 infection itself.

Our findings are consistent with previous reviews by Jackson et al. (2024), Rizzo et al. (2025) and Hessami et al. (2022), which found no clear evidence of associations between prenatal SARS-CoV-2 exposure and adverse neurodevelopment in children aged up to 12 months (Hessami et al., 2022; Jackson et al., 2024a; Rizzo et al., 2025). In contrast, our findings diverge from Pinheiro et al. (2023), which found a significant association between maternal exposure to SARS-CoV-2 during pregnancy and lower scores on fine motor and problem-solving domains (Pinheiro et al., 2023). This inconsistency is likely due to the fact that Pinheiro et al. (2023) only included studies using ASQ-3 outcomes, whereas our analysis incorporated 11 studies, only three of which used the ASQ-3, enabling a broader and more comprehensive evaluation. Similarly, our findings differ from Sturrock et al. (2023), which suggested adverse social-emotional outcomes among children prenatally exposed to SARS-CoV-2 (Sturrock et al., 2023); however, that review was only based on the results of two studies compared with our larger evidence base of 11 studies.

Our secondary analysis indicated that first trimester exposure may be associated with poorer neurodevelopmental outcomes compared with second or third trimester exposure. This conflicts with previous research on other infection types, which has shown that prenatal exposure to bacterial, viral and other infections in the second and third trimester are associated with childhood neurodevelopmental outcomes (Hardie et al., 2025a; Kwok et al., 2022). Our findings on this should be treated with particular caution as it is based on very limited evidence from three studies only (Shuffrey et al. (2022) (Shuffrey et al., 2022), Firestein et al. (2023) (Firestein et al., 2023a), and Ayesa-Arriola et al. (2023) (Ayesa-Arriola et al., 2023) with variable sample sizes and child assessment ages. Importantly, we were unable to include trimester-specific analysis conducted by Hardie et al. (2025) (Hardie et al., 2025a) in our meta-analysis due to incomparability of data (given that Hardie et al. (2025) had a binary outcome measure). Had we been able to include this it may have changed our results given no differences were observed in associations by trimester (Hardie et al., 2025a).

A key strength of our study was that we were able to incorporate a broad range of studies by standardising multiple assessment methods. However, our study does have some limitations, which are important to note. Firstly, our study's search strategy included the use of only two databases (PubMed and PsychInfo), and the omission of others (such as Cochrane, Scopus or EMBASE) may have limited the comprehensiveness of our study selection. Secondly, though our use of multiple assessment tools allowed us to include more studies, the downside of this is that this makes our findings more complicated to interpret and provides a less cohesive and more generalised picture of associations between prenatal exposure to SARS-CoV-2 infections and neurodevelopmental outcomes. Recent analysis has highlighted discrepancies in the diagnoses of developmental delays when comparing tools (Balasubramanian et al., 2024). Such variability should be considered when interpreting our results. Thirdly, a further limitation is that our study was only able to look at overall neurodevelopmental outcomes, with no domain-specific analysis. Finally, as noted above, our trimester-specific analysis was limited to just three studies with high levels of heterogeneity between studies.

## 5 Conclusion

To conclude, our findings add to previous meta-analyses and suggests that maternal SARS-CoV-2 infection during pregnancy is not associated with early childhood neurodevelopmental outcomes. While this evidence is reassuring, neurodevelopmental concerns often become more apparent as children get older (for example, when they enter formal schooling and more demands are placed on them), meaning it is important that such associations continue to be monitored longitudinally in future research as this cohort of children age and more data becomes available. Moreover, as our analysis of exposure by trimester was limited to three studies with high heterogeneity, future research should, where possible, provide more detailed assessment of timing of exposure to clarify whether risks vary by gestational age.

## CRediT authorship contribution statement

**Ryan Whalley:** Writing – original draft, Methodology, Investigation, Formal analysis, Data curation. **Amalie Primdal:** Writing – original draft, Methodology, Investigation, Formal analysis, Data curation. **Iain Hardie:** Writing – review & editing, Supervision, Methodology, Conceptualization. **Zhuoni Xiao:** Writing – review & editing, Supervision, Methodology. **Bonnie Auyeung:** Writing – review & editing, Supervision, Methodology, Conceptualization.

## Declaration of competing interest

The authors declare that they have no known competing financial interests or personal relationships that could have appeared to influence the work reported in this paper.

## Data Availability

Data will be made available on request.
