# Supplementary material for: Maternal SARS-CoV-2 infection during pregnancy and child neurodevelopmental outcomes: A systematic review and meta-analysis of observational studies
Source: Prev Med Rep. 2026 May 23;67:103508. doi: 10.1016/j.pmedr.2026.103508 (PMC13233804; doi:10.1016/j.pmedr.2026.103508)
Supplement: Supplementary file 1 — Supplementary material [file mmc1.docx]

**Supplementary Appendices**

# **Supplementary Appendix A: PRISMA 2020 checklist**

| **Section and Topic** | **Item #** | **Checklist item** | **Location where item is reported** |
| --- | --- | --- | --- |
| **TITLE** | | |  |
| Title | 1 | Identify the report as a systematic review. | Title (p1) |
| **ABSTRACT** | | |  |
| Abstract | 2 | See the PRISMA 2020 for Abstracts checklist. | Adhered to in abstract (p1) |
| **INTRODUCTION** | | |  |
| Rationale | 3 | Describe the rationale for the review in the context of existing knowledge. | Introduction section (p2-3) |
| Objectives | 4 | Provide an explicit statement of the objective(s) or question(s) the review addresses. | Research question in Introduction section (p3) |
| **METHODS** | | |  |
| Eligibility criteria | 5 | Specify the inclusion and exclusion criteria for the review and how studies were grouped for the syntheses. | Eligibility criteria section (p4) |
| Information sources | 6 | Specify all databases, registers, websites, organisations, reference lists and other sources searched or consulted to identify studies. Specify the date when each source was last searched or consulted. | Search strategy section (p4) |
| Search strategy | 7 | Present the full search strategies for all databases, registers and websites, including any filters and limits used. | Search strategy section (p4) |
| Selection process | 8 | Specify the methods used to decide whether a study met the inclusion criteria of the review, including how many reviewers screened each record and each report retrieved, whether they worked independently, and if applicable, details of automation tools used in the process. | Eligibility criteria section (p4) and Study selection process section (p4-5) |
| Data collection process | 9 | Specify the methods used to collect data from reports, including how many reviewers collected data from each report, whether they worked independently, any processes for obtaining or confirming data from study investigators, and if applicable, details of automation tools used in the process. | Data extraction and quality assessment section (p5) |
| Data items | 10a | List and define all outcomes for which data were sought. Specify whether all results that were compatible with each outcome domain in each study were sought (e.g. for all measures, time points, analyses), and if not, the methods used to decide which results to collect. | Outcome and exposure measures section (p4) |
|  | 10b | List and define all other variables for which data were sought (e.g. participant and intervention characteristics, funding sources). Describe any assumptions made about any missing or unclear information. | Outcome and exposure measures section (p4) |
| Study risk of bias assessment | 11 | Specify the methods used to assess risk of bias in the included studies, including details of the tool(s) used, how many reviewers assessed each study and whether they worked independently, and if applicable, details of automation tools used in the process. | Data extraction and quality assessment section (p5) |
| Effect measures | 12 | Specify for each outcome the effect measure(s) (e.g. risk ratio, mean difference) used in the synthesis or presentation of results. | Statistical analysis section (p5-6) |
| Synthesis methods | 13a | Describe the processes used to decide which studies were eligible for each synthesis (e.g. tabulating the study intervention characteristics and comparing against the planned groups for each synthesis (item #5)). | Statistical analysis section (p5-6) |
|  | 13b | Describe any methods required to prepare the data for presentation or synthesis, such as handling of missing summary statistics, or data conversions. | Statistical analysis section (p5-6) |
|  | 13c | Describe any methods used to tabulate or visually display results of individual studies and syntheses. | Statistical analysis section (p5-6) |
|  | 13d | Describe any methods used to synthesize results and provide a rationale for the choice(s). If meta-analysis was performed, describe the model(s), method(s) to identify the presence and extent of statistical heterogeneity, and software package(s) used. | Statistical analysis section (p5-6) |
|  | 13e | Describe any methods used to explore possible causes of heterogeneity among study results (e.g. subgroup analysis, meta-regression). | Statistical analysis section (p5-6) |
|  | 13f | Describe any sensitivity analyses conducted to assess robustness of the synthesized results. | Statistical analysis section (p5-6) |
| Reporting bias assessment | 14 | Describe any methods used to assess risk of bias due to missing results in a synthesis (arising from reporting biases). | Statistical analysis section (p5-6) |
| Certainty assessment | 15 | Describe any methods used to assess certainty (or confidence) in the body of evidence for an outcome. | Statistical analysis section (p5-6) |
| **RESULTS** | | |  |
| Study selection | 16a | Describe the results of the search and selection process, from the number of records identified in the search to the number of studies included in the review, ideally using a flow diagram. | Study selection process section (p6) and Figure 1. |
|  | 16b | Cite studies that might appear to meet the inclusion criteria, but which were excluded, and explain why they were excluded. | Study selection process section (p6) |
| Study characteristics | 17 | Cite each included study and present its characteristics. | Study characteristics section (p6) and Table 1 |
| Risk of bias in studies | 18 | Present assessments of risk of bias for each included study. | Study characteristics section (p6-7) |
| Results of individual studies | 19 | For all outcomes, present, for each study: (a) summary statistics for each group (where appropriate) and (b) an effect estimate and its precision (e.g. confidence/credible interval), ideally using structured tables or plots. | Primary and Secondary meta analysis sections (p7-8) |
| Results of syntheses | 20a | For each synthesis, briefly summarise the characteristics and risk of bias among contributing studies. | Primary and Secondary meta analysis sections (p7-8) |
|  | 20b | Present results of all statistical syntheses conducted. If meta-analysis was done, present for each the summary estimate and its precision (e.g. confidence/credible interval) and measures of statistical heterogeneity. If comparing groups, describe the direction of the effect. | Primary and Secondary meta analysis sections (p7-8) |
|  | 20c | Present results of all investigations of possible causes of heterogeneity among study results. | Primary and Secondary meta analysis sections (p7-8) |
|  | 20d | Present results of all sensitivity analyses conducted to assess the robustness of the synthesized results. | Primary and Secondary meta analysis sections (p7-8) |
| Reporting biases | 21 | Present assessments of risk of bias due to missing results (arising from reporting biases) for each synthesis assessed. | Primary and Secondary meta analysis sections (p7-8) |
| Certainty of evidence | 22 | Present assessments of certainty (or confidence) in the body of evidence for each outcome assessed. | Primary and Secondary meta analysis sections (p7-8) |
| **DISCUSSION** | | |  |
| Discussion | 23a | Provide a general interpretation of the results in the context of other evidence. | Discussion section (p8-10) |
|  | 23b | Discuss any limitations of the evidence included in the review. | Primary and Secondary meta analysis sections (p7-8) |
|  | 23c | Discuss any limitations of the review processes used. | Primary and Secondary meta analysis sections (p7-8) |
|  | 23d | Discuss implications of the results for practice, policy, and future research. | Primary and Secondary meta analysis sections (p7-8) |
| **OTHER INFORMATION** | | |  |
| Registration and protocol | 24a | Provide registration information for the review, including register name and registration number, or state that the review was not registered. | Registration section (p3) |
|  | 24b | Indicate where the review protocol can be accessed, or state that a protocol was not prepared. | Registration section (p3) |
|  | 24c | Describe and explain any amendments to information provided at registration or in the protocol. | N/a |
| Support | 25 | Describe sources of financial or non-financial support for the review, and the role of the funders or sponsors in the review. | Acknowledgements section (p10) |
| Competing interests | 26 | Declare any competing interests of review authors. | Declaration of interests section (p10) |
| Availability of data, code and other materials | 27 | Report which of the following are publicly available and where they can be found: template data collection forms; data extracted from included studies; data used for all analyses; analytic code; any other materials used in the review. | Methods section (p3-6) |

# **Supplementary Appendix B: PubMed and PsychInfo search criteria**

**PubMed Search Criteria**

("SARS-CoV-2"[Title/Abstract] OR "COVID-19"[Title/Abstract]) AND ("infection"[Title/Abstract]) AND ("pregnancy"[Title/Abstract] OR "pregnant"[Title/Abstract] OR "prenatal"[Title/Abstract]) AND ("mothers"[Title/Abstract] OR "maternal"[Title/Abstract]) AND ("neuro*"[Title/Abstract] OR "develop*"[Title/Abstract]) AND ("infant"[Title/Abstract] OR "child"[Title/Abstract] OR "offspring"[Title/Abstract])

**PsychInfo Search Criteria**

1 Prenatal.ti,ab. (22287)

2 limit 1 to peer reviewed journal (18542)

3 Maternal.ti,ab. (67471)

4 limit 3 to peer reviewed journal (55820)

5 COVID*.ti,ab. (52085)

6 limit 5 to peer reviewed journal (46674)

7 SARS*.ti,ab. (3649)

8 limit 7 to peer reviewed journal (3441)

9 Infant*.ti,ab. (90955)

10 limit 9 to peer reviewed journal (70406)

11 offspring*.ti,ab. (24119)

12 limit 11 to peer reviewed journal (20813)

15 Develop*.ti,ab. (1320397)

16 limit 15 to peer reviewed journal (946358)

17 neuro*.ti,ab. (600911)

18 limit 17 to peer reviewed journal (503535)

19 6 or 8 (47588)

20 2 or 4 (68246)

21 10 or 12 (89469)

22 16 or 18 (1327905)

23 19 and 20 and 21 and 22 (84)

24 limit 23 to english language (73)

25 limit 24 to human (69)

# **Supplementary Appendix C: Newcastle-Ottowa Scale for cohort studies**

The Newcastle-Ottawa Scale quality instrument is scored by awarding a point for each answer that is marked with an asterisk below. Possible total points are 4 points for Selection, 2 points for Comparability, and 3 points for Outcomes.

**SELECTION**

1. **Representativeness of the Exposed Cohort**
   1. Truly representative of the average patient with mental illness (*eg*, severity of illness, comorbidities) in the community*
   2. Somewhat representative of the average (*eg*, severity of illness, comorbidities)in the community*
   3. Selected group of users eg HIV+, pregnant, elderly, significant physical disabilities
   4. No description of the derivation of the cohort
2. **Selection of the Non-Exposed Cohort**
   1. Drawn from the same community as the exposed cohort*
   2. Drawn from a different source
   3. No description of the derivation of the non-exposed cohort
3. **Ascertainment of Exposure**
   1. Secure record (*eg*, medical records)*
   2. Structured interview *
   3. Written self-report
   4. No description
4. **Demonstration that Outcome of Interest Was Not Present at Start of Study**
   1. Yes*
   2. No

**COMPARABILITY**

1. **Comparability of Cohorts on the Basis of the Design or Analysis**
   1. Study controls for SES (or some reasonable proxy of SES), age, race, gender*
   2. Study controls for any additional factor* (this criteria could be modified to indicate specific control for a second important factor)
   3. Inadequate degree of control

**OUTCOME**

1. **Assessment of Outcome**
   1. Independent or blind assessment stated in the paper, or confirmation of the outcome by reference to secure records (x-rays, medical records, etc)*
   2. Record linkage (*eg*, identified through ICD codes on database records)*
   3. Self-report (*ie*, no reference to original medical records or x-rays to confirm the outcome)
   4. No description
2. **Was Follow-up Long Enough for Outcomes to Occur?**
   1. Yes (select an adequate follow up period for outcome of interest)*
   2. No
3. **Adequacy of Follow-up of Cohorts**
   1. Complete follow-up—all subjects accounted for*
   2. Subjects lost to follow-up unlikely to introduce bias—small number lost (LESS than 20% follow-up, or description provided of those lost)*
   3. Follow-up rate MORE than 20% and no description of those lost
   4. No statement

Thresholds for converting the Newcastle-Ottawa scales to AHRQ standards (good, fair, and poor):

- Good quality: 3 or 4 stars in selection domain AND 1 or 2 stars in comparability domain AND 2 or 3 stars in outcome/exposure domain
- Fair quality: 2 stars in selection domain AND 1 or 2 stars in comparability domain AND 2 or 3 stars in outcome/exposure domain
- Poor quality: 0 or 1 star in selection domain OR 0 stars in comparability domain OR 0 or 1 stars in outcome/exposure domain

Source: Gierisch JM, Beadles C, Shapiro A, et al. Health Disparities in Quality Indicators of Healthcare Among Adults with Mental Illness [Internet]. Washington (DC): Department of Veterans Affairs (US); 2014 Oct. APPENDIX B, NEWCASTLE-OTTAWA SCALE CODING MANUAL FOR COHORT STUDIES. Available from: <https://www.ncbi.nlm.nih.gov/books/NBK299087/>

# **Supplementary Appendix D: NOS quality assessment scores across selection, comparability and outcome**

| **Study authors and year** | **NOS selection score (/4)** | **NOS comparability score (/2)** | **NOS outcome score (/3)** | **NOS total score (/9)** |
| --- | --- | --- | --- | --- |
| Ayesa-Arriola et al (2023) | 3 | 1 | 3 | 7 |
| Edlow et al. (2022) | 4 | 2 | 2 | 8 |
| Edlow et al. (2023) | 4 | 2 | 3 | 9 |
| Fajardo-Martinez et al. (2024) | 3 | 1 | 3 | 7 |
| Firestein et al. (2023) | 2 | 1 | 3 | 7 |
| Firestein et al. (2024a) | 3 | 2 | 3 | 8 |
| Firestein et al. (2024b) | 3 | 2 | 3 | 8 |
| Hardie et al. (2025) | 4 | 2 | 2 | 8 |
| Jackson et al. (2024) | 3 | 2 | 2 | 7 |
| Jaswa et al. (2024) | 4 | 2 | 2 | 8 |
| Santos et al. (2024) | 4 | 1 | 2 | 7 |
| Shuffrey et al. (2022) | 4 | 1 | 1 | 6 |

# **
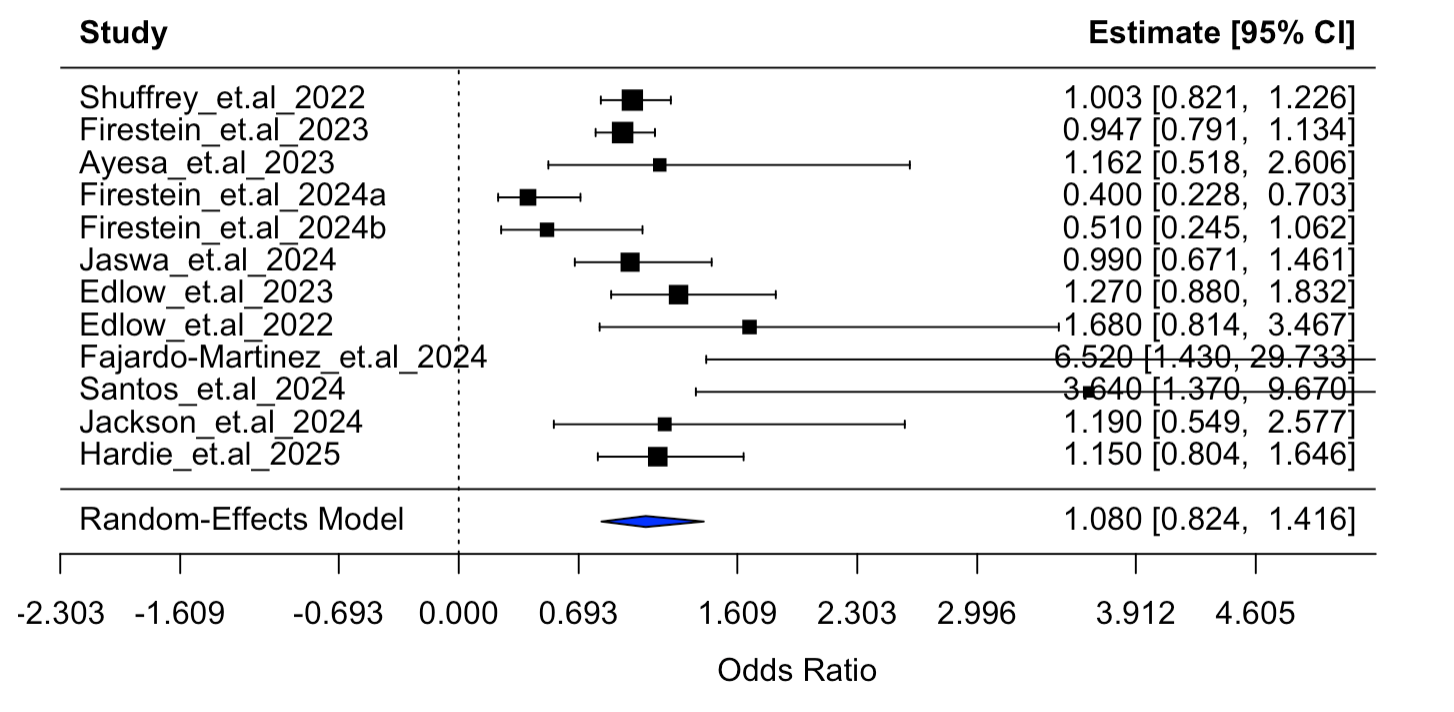
Supplementary Appendix E: Forest plot for primary meta-analysis**

# **Supplementary Appendix F: Funnel plot assessing potential publication bias in individual studies (high-variance studies included)**


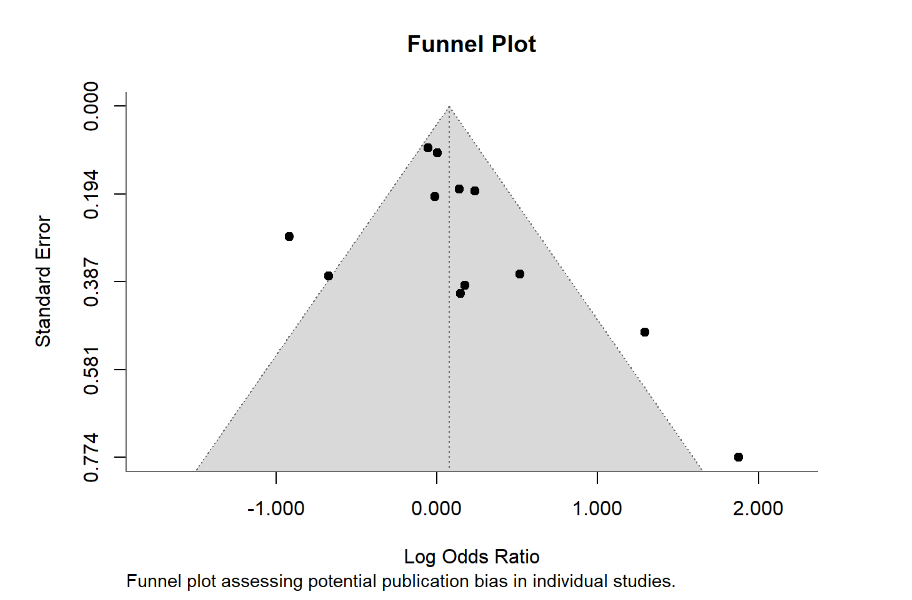


# **Supplementary Appendix G: Funnel plot assessing potential publication bias in individual studies (high-variance studies excluded)**

**
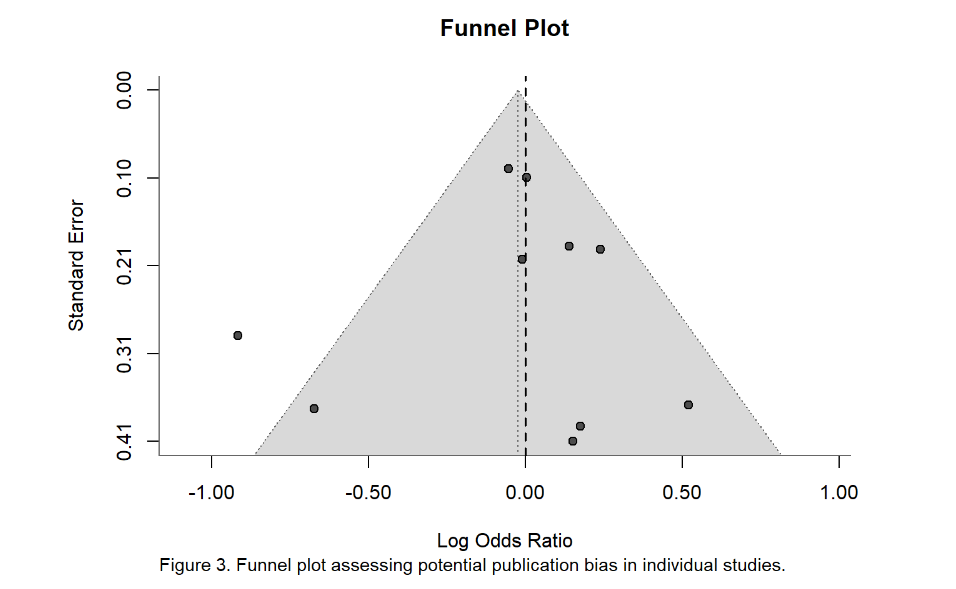
**
